# A Task-Learning Strategy for Robotic Assembly Tasks from Human Demonstrations

**DOI:** 10.3390/s20195505

**Published:** 2020-09-25

**Authors:** Guanwen Ding, Yubin Liu, Xizhe Zang, Xuehe Zhang, Gangfeng Liu, Jie Zhao

**Affiliations:** State Key Laboratory of Robotics and System, Harbin Institute of Technology, Harbin 150001, China; 17B908042@stu.hit.edu.cn (G.D.); liuyubin@hit.edu.cn (Y.L.); zhangxuehe@hit.edu.cn (X.Z.); liugangfeng@hit.edu.cn (G.L.); jzhao@hit.edu.cn (J.Z.)

**Keywords:** dynamic movement primitives, human–robot skills transfer, movement segmentation, robotic assembly, visuo-spatial skill learning

## Abstract

In manufacturing, traditional task pre-programming methods limit the efficiency of human–robot skill transfer. This paper proposes a novel task-learning strategy, enabling robots to learn skills from human demonstrations flexibly and generalize skills under new task situations. Specifically, we establish a markerless vision capture system to acquire continuous human hand movements and develop a threshold-based heuristic segmentation algorithm to segment the complete movements into different movement primitives (MPs) which encode human hand movements with task-oriented models. For movement primitive learning, we adopt a Gaussian mixture model and Gaussian mixture regression (GMM-GMR) to extract the optimal trajectory encapsulating sufficient human features and utilize dynamical movement primitives (DMPs) to learn for trajectory generalization. In addition, we propose an improved visuo-spatial skill learning (VSL) algorithm to learn goal configurations concerning spatial relationships between task-relevant objects. Only one multioperation demonstration is required for learning, and robots can generalize goal configurations under new task situations following the task execution order from demonstration. A series of peg-in-hole experiments demonstrate that the proposed task-learning strategy can obtain exact pick-and-place points and generate smooth human-like trajectories, verifying the effectiveness of the proposed strategy.

## 1. Introduction

**Figure 1:**
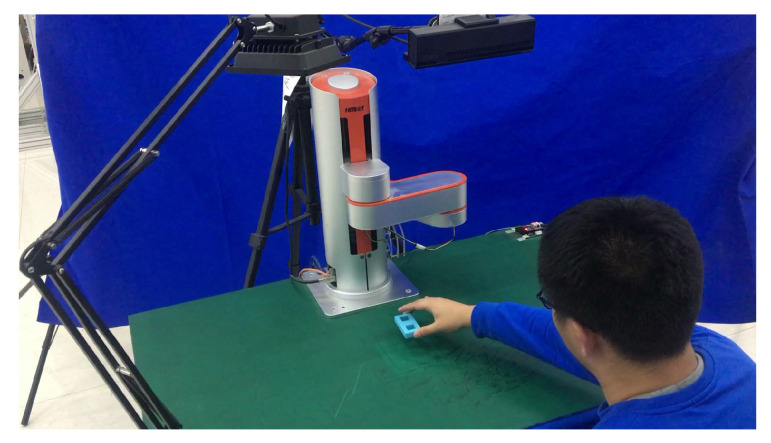
Close-up figure of Learning from Demonstration (LfD) with a human teaching the robot through demonstration.

Recent advances in artificial intelligence and sensor technology have heightened the need for robots to perform assembly tasks autonomously. However, the applications in manufacturing remain a significant challenge, since traditional industrial robots deployed in production lines are pre-programmed for a specific task in a carefully structured environment. To overcome these challenges on different levels, the field of industrial robotics is now moving towards Human–Robot Collaboration (HRC) [1,2,3,4,5,6,7]. In the area of HRC, Robot Learning from Demonstration (LfD) [8] provides a natural and intuitive mechanism for humans to teach robots new skills without relying on professional knowledge. Robots first observe human demonstrations, then extract task-relevant features, derive the optimal policy between the world states and actions, finally reproduce and generalize tasks in different situations, and refine policy during practice [9] (See Figure 1).

Given that most current robotic assembly tasks in LfD [10,11,12] are demonstrated by human hands, capturing human hand movements makes it a crucial step for robots to understand human intentions. Human hand movements are often treated as trajectories with capturing methods categorized into kinesthetic demonstration [11], motion-sensor demonstration [13,14], and teleoperated demonstration [15]. In kinesthetic demonstration, robots are guided by humans directly, without tackling correspondence problems owing to different kinematics and dynamics between each other [13]. However, it relies heavily on the cooperative ability of industrial manipulators and fails when facing heavy objects or dangerous environments. Motion-sensor demonstration usually utilizes external markers [13] or special equipment [14], exhibiting flexibility compared to kinesthetic demonstration. Nevertheless, it relies on the illumination conditions and brings out correspondence problems when considering gesture imitation problems [13]. Teleoperated demonstration, similar to kinesthetic demonstration, constructs a real-time interaction interface between humans and robots. Unfortunately, it relies on the teleoperation ability of the manipulators at an expensive cost. To overcome drawbacks mentioned above, research in [16] has provided a promising path for capturing human hand movements by introducing a state-of-the-art Kinect FORTH system [17], which is markerless and relatively insensitive to illumination conditions, obtaining the continuous and accurate poses of human hand articulations in near real-time. However, when facing occlusions with the human hand, the method has a poor performance. In this paper, based upon the Kinect FORTH system, we establish a vision capture system to acquire continuous human hand movements during the demonstration.

To encode human hand movements with task-oriented models, movement primitives (MPs) [18] are well-established methods in robotics. Generally, movement primitive learning methods fall into two groups: one is based on probabilistic models [11,19,20], the other is based on dynamical systems [21,22]. Probabilistic models commonly take the form of a Hidden Markov Model and Gaussian Mixture Regression (HMM-GMR) [19], Gaussian Mixture Model and Gaussian Mixture Regression (GMM-GMR) [11]. They use HMM and GMM to model joint probability distributions of multiple demonstration data and derive the desired trajectory using GMR. However, both HMM-GMR and GMM-GMR methods cannot adapt to new task situations. Recently, the Task-parametrized Gaussian Mixture Models (TP-GMMs) proposed in [20] encapsulate task parameters into GMMs for adaptation to new situations encountered in robotic tasks. Nevertheless, TP-GMMs will fail when task parameters do not appear in the model during the learning process. Dynamical movement primitives (DMPs) [21] are well-investigated methods under the category of dynamical systems. DMPs describe dynamical systems with a series of nonlinear differential equations equivalent to a linear spring system modulated by a nonlinear external force. However, DMPs only use one demonstration for learning which ones have suboptimal model parameters. The work [22] proposes a combination method of Reinforcement Learning (RL) and DMPs to learn couplings across different control variables. Apart from two categories, the Mixture of Motor Primitives (MoMPs) [23] and Probabilistic Movement Primitives (ProMPs) [24] methods are new frameworks proposed in movement primitives learning. MoMPs use a gating network based on augmented states to activate different movement primitives and use mixed weights to generate new movements under new task situations. ProMPs represent movement primitives by hierarchical Bayesian models and generate movements with probability inference. Compared to DMPs, ProMPs can achieve this via-point generalization rather than start-points and end-points generalization in DMPs. However, ProMPs need a greater number of demonstrations to obtain reliable trajectory distributions. In this paper, since we tend to accomplish peg-in-hole tasks by generalizing start-points and end-points rather than via-points, movement primitives learning is achieved using DMPs. We adopt GMM-GMR to extract the optimal trajectory from multiple human demonstrations, then passed to DMPs for learning.

For multistep robotic assembly tasks like peg-in-hole [25] and chair assembly [26,27], encoding whole movements with one movement primitive is unrealistic. Whole movements are supposed to be represented by a sequence of movement primitives explaining human demonstrations, and after movement segmentation, robots can reuse and reorder the same set of movement primitives to accomplish similar but different robotic tasks without external demonstrations. Previous work in movement segmentation including methods based on heuristic [12] and unsupervised learning [26,27]. Heuristic-based methods utilize the prior knowledge of robot kinematic and dynamics properties. In [12], zero-crossing velocity (ZCV) is used for movement segmentation in robotic assembly tasks. However, ZCV usually leads to sub-optimal segmentation results. In contrast, unsupervised learning methods cluster similar movements with minimum prior knowledge. HMMs are popular methods in this context. However, the number of unobservable states needs to be chosen in advance. To tackle the problems, recent work in [26] has used the Beta Process Autoregressive HMM (BP-AR-HMM) algorithm [28] with potentially infinite states, which can be shared and switched between each other. Further, in [27], the Probabilistic Segmentation (ProbS) algorithm is proposed to improve a given segmentation concurrently through the Expectation Maximum (EM) [29] algorithm. Nevertheless, both BP-AR-HMM and ProbS need a time-consuming iterative procedure, reducing segmentation efficiency. In this paper, we propose a threshold-based heuristic segmentation algorithm to segment complete movements into different movement primitives automatically. Compared to ZCV [12], we can obtain better segmentation results and achieve fast segmentation with no need for a large amount of training time compared to BP-AR-HMM [26].

Despite learning movement primitives from human demonstrations, goal configuration learning [30,31,32] concerning spatial relationships between task-relevant objects need to be considered. The work in [31] proposes a novel Visuospatial Skill Learning (VSL) method with the ability to learn from observing human demonstrations directly with only visual inputs. VSL learns goal configurations from only one multioperation demonstration with minimum prior knowledge and generalizes the learned goal configurations under new situations maintaining the task execution order. Trajectory-generation with this method is equation-based, and further research in [32] combines DMPs to acquire human-like trajectories. However, VSL will sometimes fail when occlusions occur during the task execution process. For instance, in robotic jigsaw tasks [31], after the robot picks and places the first jigsaw based on the generalized goal configurations in advance, then it will determine the next pick-and-place points for the second jigsaw during the task execution process. If task-relevant objects are occluded by the robot body, the VSL method will not find accurate pick-and-place points. In this paper, aiming at fundamental peg-in-hole tasks, we propose an improved VSL algorithm with modifications to [32] by defining peg-in-hole observations and offsets to learn goal configurations from observing human demonstration directly. Only one complete multioperation demonstration is required for learning, and robots can generalize goal configurations under new task situations following the task execution order. Through the improved VSL algorithm, we can generalize all the actual 3D pick-and-place points with only one image captured before the robot executes peg-in-hole tasks, and the peg-in-hole objects and base are fixed during the task execution process, so the occlusion problems will not occur.

**Figure 2:**
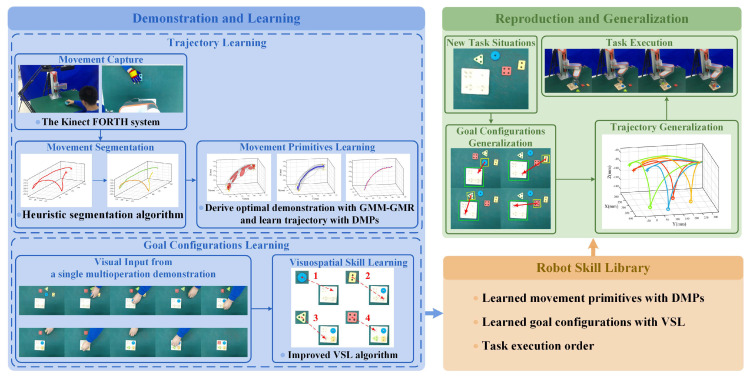
The overall proposed task-learning strategy of peg-in-hole tasks.

A workflow diagram of the proposed task-learning strategy can be seen in Figure 2. The strategy consists of two phases: the demonstration and learning phase, and the reproduction and generalization phase, with the robot skill library conducting their interactions.

During the demonstration and learning phase, we demonstrate pick-and-place operations of a blue object multiple times as basic trajectory components in peg-in-hole tasks. We track human hand centroid movements by the Kinect FORTH system and segment complete movements into three phases represented by different movement primitives using the proposed heuristic segmentation algorithm. For each segmented movement primitive, we adopt GMM-GMR to derive the optimal trajectory from multiple demonstrations, learn with DMPs and store into the robot skill library for trajectory generalization. Subsequently, we record the video of a single multioperation demonstration in peg-in-hole tasks and use the improved VSL algorithm for goal configuration learning. The task execution order and learned goal configurations from the demonstration are stored into the robot skill library for goal configurations generalization.

During the reproduction and generalization phase, the robot reproduces and generalizes different peg-in-hole tasks under new task situations with images captured under the Kinect view. Based upon previously learned goal configurations, we can obtain actual pick-and-place points through the improved VSL algorithm, then pass them to DMPs for trajectory generalization. Trajectory segments of each phase are finally connected to complete trajectories following the task execution order for robots to accomplish peg-in-hole tasks.

To summarize, the main contributions of this paper are as follows:

1. We establish a markerless vision capture system based on the Kinect FORTH System to track continuous human hand centroid movements and develop a threshold-based heuristic movement segmentation algorithm to segment human hand movements into different movement primitives automatically with no need for a large amount of training time.
2. We adopt the GMM-GMR method to derive the optimal trajectory encapsulating sufficient human features and utilize DMPs to learn for trajectory generalization, the generated trajectories exhibit satisfactory smoothness and similarity with respect to human demonstrations.
3. We propose an improved VSL algorithm enabling robots to learn goal configurations directly from the video of only one multioperation demonstration and generalize them under new task situations, the generated goal configurations reflect the actual pick-and-place points of peg-in-hole tasks exactly.

The remainder of this paper is organized as follows. Section 2 details the overall task-learning methodology, and Section 3 presents the experiment study for a series of peg-in-hole robotic assembly tasks. Section 4 analyzes the experimental results and summarizes the advantages and disadvantages of the proposed strategy, and finally, we conclude the paper and discuss future directions in Section 5.

## 2. Methodology

The emphasis of this paper lies in allowing robots to learn and generalize robotic assembly tasks autonomously from human demonstrations, without taking into account gesture imitation [13] and obstacle avoidance problems. Hence, we assume that the learned human hand movements can directly apply to the end-effector, and task-relevant objects involved will not collide and interfere with each other during task execution. The orientation of the end-effector is fixed, using XYZ positions to accomplish peg-in-hole tasks.

### 2.1. Data Acquisition and Preprocessing from Markerless Demonstrations

The Kinect FORTH system [17] merely relies on markerless visual data acquired from the Kinect sensor to track 3D positions, orientations and full articulations of a human hand robustly and accurately. In this work, we choose human hand centroid point as the tracking point with trajectory coordinate sets ${\left\{x_{t,i},y_{t,i},z_{t,i}\right\}}_{i=1}^{N}$ from *N* demonstrations stored as the original data. The trajectory data are then transformed to the base frame of the robot based on hand-eye calibration. To reduce the noise generated from illuminations and occlusions of the vision capture system, we apply a Moving Average Filter (MAF) with a window width *n* set experimentally to ensure satisfactory smoothness and minimum distortion loss of trajectories. Multiple demonstrations usually have different timesteps, we thus utilize Cubic Spline Interpolation (CSI) to normalize trajectories to the same timesteps.

### 2.2. Heuristic Movement Segmentation Algorithm

**Figure 3:**
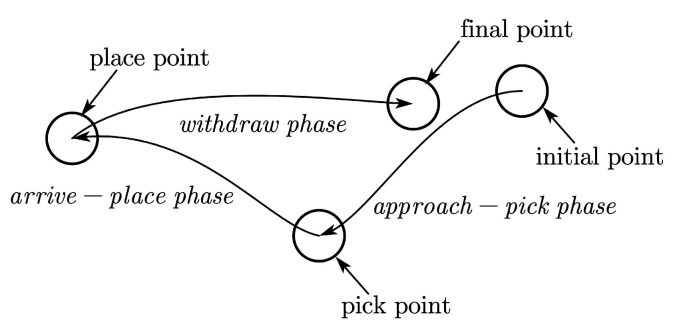
Segmentation process of basic pick-and-place operations. Points “O” represent segmentation points with arrows indicating the operation sequence.

Generally, we represent whole human hand movements in basic pick-and-place operations of peg-in-hole tasks with three phases, as shown in Figure 3.

1. The approach-pick phase, during which the human moves his hand from an initial point, approaches a pick point just before he makes contact with the object and grasps it.
2. The arrive-place phase, during which the human grasps the object from a pick point and arrives at a place point just before he releases the object.
3. The withdraw phase, during which the human releases the object from a place point and returns to a final point.

Inspired by research on vision-based action recognition [33] that possible Grasp and Release points may occur at the local lowest points of human hand palm motions, we propose a heuristic segmentation algorithm based on human hand centroid velocities, as depicted in Algorithm 1.
**Algorithm 1** Heuristic Movement Segmentation Algorithm.**Input:** Human hand centroid trajectory coordinates  ${\left\{x_{t,i},y_{t,i},z_{t,i}\right\}}_{i=1}^{N}$**Output:** Segmentation points set of pick-and-place operations  $S={\left\{S_{approach-pick,i},S_{arrive-place,i},S_{withdraw,i}\right\}}_{i=1}^{N}$  1:**for** i=1 to *N* **do**  2: **for** each Cartesian dimension $j\in \left(1,2,3\right)$ **do**  3:  Calculate velocities by the differential process;  4:  Smooth velocities using MAF twice and normalize velocities using CSI;  5: **end for**
  6: Calculate the square sum of velocities in all Cartesian dimensions and normalize the results   ${\left\{v_{t,i}\right\}}_{i=1}^{N}$ within the range $\left[0,1\right]$;  7: **Initialization:** $S_{approach-pick,i}=S_{arrive-place,i}=S_{withdraw,i}=S_{min,i}=S_{int,i}=S_{distmin-index,i}=\emptyset$  8: $S_{min,i}=FindLocalMins\left(v_{t,i},t_{start},t_{end}\right);$
  9: $S_{min,i}=ThresholdElimination\left(S_{min,i},\vartheta \right);$
10: $S_{int,i}=FindIntersection\left(v_{t,i},\vartheta \right);$
11: **if** $∥S_{int,i}\left(1\right)-t_{start}∥\;\;<\;\;∥S_{int,i}\left(1\right)-S_{min,i}\left(1\right)∥$ **then**12:  $S_{int,i}=DeleteFirstIndex\left(S_{int,i}\right)$;13: **end if**14: $S_{distmin-index,i}=FindMinIndex\left(S_{int,i},S_{min,i}\right);$
15: $S_{approach-pick,i}=\left[t_{start}:t_{index\_first,i}\right];$
16: $S_{arrive-place,i}=\left[t_{index\_second,i}:t_{index\_third,i}\right];$
17: **if** $Length\left(S_{distmin-index,i}\right)<5$ **then**18:  $S_{withdraw,i}=\left[t_{index\_fourth,i}:t_{end}\right];$
19: **else**
20:  **if** $t_{int\_last,i}>t_{index\_last,i}$ **then**
21:   $S_{withdraw,i}=\left[t_{index\_fourth,i}:t_{int\_last,i}\right];$
22:  **else**
23:   $S_{withdraw,i}=\left[t_{index\_fourth,i}:t_{index\_last,i}\right]$;24:  **end if**
25: **end if**
26:**end for**27:**Return:**  $S={\left\{S_{approach-pick,i},S_{arrive-place,i},S_{withdraw,i}\right\}}_{i=1}^{N}$


For each trajectory in *N* demonstrations, we first calculate velocities in each Cartesian dimension by the differential process. Considering large velocity fluctuation during the human demonstration, we applied MAF twice to smooth velocities and normalize to the same timesteps by CSI. The velocity square sum is calculated and normalized within the range $\left[0,1\right]$.

An initialization step is then performed to establish segmentation points set $S_{approach-pick,i},\;S_{arrive-place,i},\;S_{withdraw,i}$ and auxiliary points set $S_{min,i},\;S_{int,i},\;S_{distmin-index,i}$. Considering movement switching may occur at the local minima, we find all the local minimum of velocities $v_{t,i}$ and add them into the local minimum points set $S_{min,i}$ using the FindLocalMins function with velocities $v_{t,i}$, the start point $t_{start}$ and the end point $t_{end}$ as input. To eliminate local minimum points caused by human hand quiver rather than movements switching, we determine a threshold ϑ experimentally and use the ThresholdElimination function to update the local minimum points set $S_{min,i}$ by eliminating those local minimum points whose corresponding velocities are above the threshold. Meanwhile, the FindIntersection function expands the threshold value to a line with the same timesteps as velocities and find the intersection points between each other, the points are then rounded off and added into the intersection points set $S_{int,i}$. For the first intersection point $S_{int,i}\left(1\right)$, we calculate the distance between the start point $t_{start}$ and the first local minimum point $S_{min,i}\left(1\right)$. If the distance of the former is less than the latter, we delete the first intersection point using the DeleteFirstIndex function to update the intersection points set $S_{int,i}$. That is to say, if a threshold-based intersection occurs at a moment closer to the start point, we select the start point as the first segment point.

After updating $S_{min,i}$ and $S_{int,i}$, we traverse elements in $S_{int,i}$ to calculate distances between each other, the FindMinIndex function finds the corresponding indexes of the minimum distance in $S_{min,i}$ and adds them into the minimum distance index set $S_{distmin-index,i}$. Finally, the segmentation points set can be obtained from $S_{distmin-index,i}$, $S_{int,i},t_{start},t_{end}$ based on heuristic rules. Specifically, we add $t_{start}$ and the first index $t_{index\_first,i}$ from $S_{distmin-index,i}$ to construct the approach-pick phase $S_{approach-pick,i}$. Then, the second index $t_{index\_second,i}$ and the third index $t_{index\_third,i}$ from $S_{distmin-index,i}$ are added to the arrive-place phase $S_{arrive-place}$. The withdraw phase $S_{withdraw,i}$ is constructed based on the length of $S_{distmin-index,i}$. If the length is less than 5, that is, there are no intersections in the withdraw phase, we thus add the fourth index $t_{index\_fourth,i}$ from $S_{distmin-index,i}$ and $t_{end}$ to the withdraw phase $S_{withdraw,i}$. If not, there are two cases to consider. The first case is that if the last point $t_{int\_last,i}$ of $S_{int,i}$ is greater than of the last $t_{index\_last,i}$ from $S_{distmin-index,i}$, we add the fourth index $t_{index\_fourth,i}$ from $S_{distmin-index,i}$ and the last point $t_{int\_last,i}$ of $S_{int,i}$ to $S_{withdraw,i}$. The meaning is that there is no switching of movements that occurs after the last intersection point, thus making it the end point of the withdraw phase. The other case occurs when the human quivers his hand at the end of the withdraw phase with the local minimum points detected, the fourth index $t_{index\_fourth,i}$ and the last index $t_{index\_last,i}$ are added to $S_{withdraw,i}$ as a consequence. The output segmentation points set $S={\left\{S_{approach-pick,i},S_{arrive-place,i},S_{withdraw,i}\right\}}_{i=1}^{N}$ is used to index the whole trajectories of *N* demonstrations and store them into different movement primitives separately for movement primitives learning.

### 2.3. Optimal Demonstration Trajectroy Extraction Using GMM-GMR

After segmentation, *N* trajectories in the same phase usually have different durations. We adopt the Dynamical Time Warping (DTW) [34] method for time alignment. Considering the aligned dataset $\xi ={\left\{\xi_{t},\xi_{s,i}\right\}}_{i=1}^{N}$ with *N* matrixes of 4×T dimensionality. *T* is the identical duration of multiple demonstrations after alignment, *t* is the timestep index of each demonstration and $s\in R^{3}$ is the corresponding Cartesian positions of human hand centroid. The dataset can be modelled by GMM through a mixture of *K* Gaussian components with dimensionality D=4 as follows:

(1) $$p\left(\xi \right)=\sum_{k=1}^{K}p\left(k\right)p\left(\xi |k\right)$$

(2) $$p\left(k\right)=\pi_{k}$$

(3) $$\begin{array}{cc} p\left(\xi |k\right) & =N\left(\xi ;\mu_{k},\Sigma_{k}\right)\\ \; & =\frac{1}{\sqrt{{\left(2\pi \right)}^{D}\left|\Sigma_{k}\right|}}e^{-\frac{1}{2}\left({\left(\xi -\mu_{k}\right)}^{T}\Sigma_{k}^{-1}\left(\xi -\mu_{k}\right)\right)}. \end{array}$$

The parameters $\left\{K,\pi_{k},\mu_{k},\Sigma_{k}\right\}$ of GMM define the number, prior, mean and covariance matrix of Gaussian components, respectively. We select the optimal number *K* using the BIC criteria [35]. The parameters $\left\{\pi_{k},\mu_{k},\Sigma_{k}\right\}$ of each component are estimated iteratively using the expectation-maximization (EM) algorithm [29]. EM is a method suitable for parameters estimation with hidden variables, however, it is sensitive to initial values, getting trapped into a local minimum easily. As a consequence, k-means clustering method is applied to initialize parameters for iteration.

After updating parameters $\left\{K,\pi_{k},\mu_{k},\Sigma_{k}\right\}$, we apply GMR to generate a desired trajectory. For each Gaussian component, the mean and covariance matrix are separated as follows:

(4) $$\mu_{k}=\left[\begin{array}{c} \mu_{t,k}\\ \mu_{s,k} \end{array}\right],\Sigma_{k}=\left[\begin{array}{cc} \Sigma_{t,k} & \Sigma_{ts,k}\\ \Sigma_{st,k} & \Sigma_{s,k} \end{array}\right].$$

The temporal values $\xi_{t}$ are then used as the input data to estimate the conditional expectation ${\hat{\xi }}_{s,k}$ and covariance ${\hat{\Sigma }}_{s,k}$:

(5) $$\begin{array}{cc} {\hat{\xi }}_{s,k} & =\mu_{s,k}+\Sigma_{st,k}{\left(\Sigma_{t,k}\right)}^{-1}\left(\xi_{t}-\mu_{t,k}\right)\\ {\hat{\Sigma }}_{s,k} & =\Sigma_{s,k}-\Sigma_{st,k}{\left(\Sigma_{t,k}\right)}^{-1}\Sigma_{ts,k}. \end{array}$$

The mixing coefficient $\beta_{k}$ is defined:

(6) $$\beta_{k}=\frac{p\left(k\right)p\left(\xi_{t}|k\right)}{\sum_{k=1}^{K}p\left(k\right)p\left(\xi_{t}|k\right)}$$

and the overall expectation ${\hat{\xi }}_{s}$ and covariance ${\hat{\Sigma }}_{s}$ are:

(7) $${\hat{\xi }}_{s}=\sum_{k=1}^{K}\beta_{k}{\hat{\xi }}_{s,k},{\hat{\Sigma }}_{s}=\sum_{k=1}^{K}\beta_{k}^{2}{\hat{\Sigma }}_{s,k}.$$

The datasets $\left\{\xi_{t},{\hat{\xi }}_{s}\right\}$ are treated as the extracted optimal trajectory using GMM-GMR and passed to DMPs as input for subsequent learning.

### 2.4. Trajectory Learning and Generalization Using DMPs

DMPs encode discrete and rhythmic movements with linear differential equations modulated by nonlinear functions. In this paper, we focus on discrete movements and encode each degree of freedom (DOF) in Cartesian space with a separated DMP described by the canonical system:

(8) $$\tau \dot{x}=-\alpha_{x}x$$

and the transformation system:

(9) $$\begin{array}{cc} \tau \dot{z} & =\alpha_{z}\left(\beta_{z}\left(G-y\right)-z\right)+f\\ \tau \dot{y} & =z, \end{array}$$

where τ is the time constant, *x* is the phase variable indicates the movement evolution, $\alpha_{x}$ determines the decay rate. *y* defines the position, $z,\dot{z}$ are respectively velocity and acceleration intermediate variables. $\alpha_{z}$ and $\beta_{z}$ are constants set to be critically damped. *G* is the target position of the movement.

For trajectory learning, *f* is a nonlinear function represented by a linear combination of $K^{\prime }$ normalized radial basis functions $\Psi_{i}\left(x\right)$ as follows:

(10) $$f\left(x\right)=\frac{\sum_{i=1}^{K^{\prime }}\Psi_{i}\left(x\right)w_{i}}{\sum_{i=1}^{K^{\prime }}\Psi_{i}\left(x\right)}x\left(G-y_{0}\right)$$

(11) $$\Psi_{i}\left(x\right)=\exp\left(-\frac{1}{2\sigma_{i}^{2}}{\left(x-c_{i}\right)}^{2}\right),$$

where $w_{i}$ denotes mixture weights, $y_{0}$ is the start point, $\sigma_{i}$ and $c_{i}$ are respectively the width and center of basis functions distributing along the phase variable *x* with equal spacing. The weights $w_{i}$ can be learned through the locally weighted regression (LWR) method [21].

After learning, trajectory generalization is achieved with DMPs by adapting new start position $y_{0}^{\prime }$ and target position $G^{\prime }$, then computing $f\left(x\right)$ using Equation (10) , finally integrating the transformation system using Equation (9) to generate desired trajectories for task execution.

### 2.5. Goal Configuration Learning and Generalization Using the Improved VSL Algorithm

Formally, we represent the goal configuration learning process as a tuple:

(12) $$V=\left\{\eta ,W,O,F,P,A\right\},$$

where η determines the number of pick-and-place operations involved in peg-in-hole tasks. $W=\left\{W_{D},W_{G}\right\}\in R^{V\times U}$ defines image sequences of the world under the camera view with $W_{D}$ and $W_{G}$ denote respectively the demonstration phase and the generalization phase. *U* and *V* are resolution parameters. $O=\left\{O_{Pick},O_{Peg}\right\}$ are image sequences of observations holding the essential parts of the world described by observation frames $F\in R^{V^{\prime }\times U^{\prime }}$. F determine rectangle areas in 2D images with resolution parameters $U^{\prime }$ and $V^{\prime }$. In this work, we define pre-pick observation and peg-in-hole observation sequences $O=\left\{O_{Pick},O_{Peg}\right\}$ for modifications to the original VSL method [32]. In particular, $O_{pick}$ are images captured just before the human approaches task-relevant objects and grasps them, and $O_{peg}$ are images captured after the human accomplishes each peg-in-hole operation with task-relevant objects stabilized in their own positions. The observation sequences can be obtained from the video easily and robustly to achieve accurate and stable goal configuration generalization. $P=\left\{P_{Pick},P_{Place}\right\}$ are output goal configurations of the generalization phase. In this work, goal configurations are equivalent to actual pick-and-place points of peg-in-hole tasks with A, the previously segmented and learned movement primitives set. Based upon representations above, the implementation of the improved VSL algorithm is detailed in Algorithm 2.

To initialize, $F=\left\{F_{Pick},F_{Peg}\right\}$ are set according to objects maximum size. The initial points $P_{initial}$ and the final points $P_{final}$ are set the same for each peg-in-hole operation. Meanwhile, the pick offsets $D_{Pick}$ and peg-in-hole offsets $D_{Peg}$ are set experimentally for adaption to peg-in-hole tasks. During the demonstration phase, $W_{D}$ are a sequence of pre-action and post-action images obtained via key frame extraction from the video. We utilize RecordPrePickObs and RecordPegInHoleObs functions to obtain $O_{Pick}\left(i\right)$ and $O_{peg}\left(i\right)$ respectively. Image substracting and thresholding methods are performed with morphological erosion and dilation processed to extract pixel centers and add to observation sequences under observation frames $F_{Pick}$ and $F_{Peg}$. During the generalization phase, $W_{G}$ are new task situations captured under the camera view for each operation. Subsequently, the FindBestMatch function finds best matched feature point pairs $f_{Pick}^{*}$ exploiting image matching methods. We adopt the Speeded Up Robust Features (SURF) algorithm [36] to match features along with the M-estimator Sample Consensus (MSAC) algorithm [37] to reject error matching pairs and calculate the homography matrix. Subsequently, the FindPickPoint and FindPlacePoint function transform pixel centers of $O_{Pick}\left(i\right)$ and $O_{Peg}\left(i\right)$ to those in $W_{G}$ as pick-and-place points $P_{Pick}^{*}\left(i\right),P_{Place}^{*}\left(i\right)$ under the image plane. Further, we transform those points to actual 3D pick-and-place points $P_{Pick}\left(i\right),P_{Place}\left(i\right)$ under the robot base frame using the Transform function based on hand-eye calibration with $D_{Pick}$ and $D_{Peg}$ added. Finally, based upon previously segmented and learned movement primitives as well as goal configurations generalized from previous steps, robots are able to accomplish peg-in-hole tasks under new situations using functions PickObject, PlaceObject, and Return. To illustrate the relationships between trajectory learning and goal configuration-learning from these functions, we take the PickObject function as an example and the functions PlaceObject and Return are run in the same way. Specifically, the PickObject function utilizes the initial points $P_{initial}$ set fixed for each peg-in-hole operation and the generalized pick points $P_{Pick}\left(i\right)$ for each peg-in-hole operation as query points for the trajectory learned with DMPs in the approach-pick phase (i.e., $A_{approach-pick}$) to generalize new trajectories under new task situations for each peg-in-hole operation.
**Algorithm 2** The Improved VSL Algorithm for Robotic Peg-in-Hole Tasks.**Input**:
$\left\{\eta ,W,F,A\right\}$**Output**:
$\left\{P\right\}$  1:**Initialization:**  $F=\left\{F_{Pick},F_{Peg}\right\},P_{initial},P_{final},D_{Pick},D_{Peg}$  2:\\ The Demonstration Phase  3:**for**i=1 to η **do**  4: $O_{pick}\left(i\right)=RecordPrePickObs\left(W_{D},F_{Pick}\right)$;  5: $O_{Peg}\left(i\right)=RecordPegInHoleObs\left(W_{D},F_{Peg}\right)$;  6:**end for**  7:\\ The Generalization Phase  8:**for** i=1 to η **do**  9: $f_{Pick}^{*}=FindBestMatch\left(W_{G},O_{Pick}\left(i\right)\right)$;10: $P_{Pick}^{*}\left(i\right)=FindPickPoint\left(f_{Pick}^{*}\right)$;11: $P_{Pick}\left(i\right)=Transform\left(P_{Pick}^{*}\left(i\right),D_{Pick}\right)$;12: $PickObject\left(P_{initial},P_{Pick}\left(i\right),A_{approach-pick}\right)$;13: $f_{Peg}^{*}=FindBestMatch\left(W_{G},O_{Peg}\left(i\right)\right)$;14: $P_{Place}^{*}\left(i\right)=FindPlacePoint\left(f_{Peg}^{*}\right)$;15: $P_{Place}\left(i\right)=Transform\left(P_{Place}^{*}\left(i\right),D_{peg}\right)$;16: $PlaceObject\left(P_{Pick}\left(i\right),P_{Place}\left(i\right),A_{arrive-place}\right)$;17: $Return\left(P_{Place}\left(i\right),P_{final},A_{withdraw}\right)$;18:**end for**

## 3. Experimental Study

### 3.1. Experimental Setup

**Figure 4:**
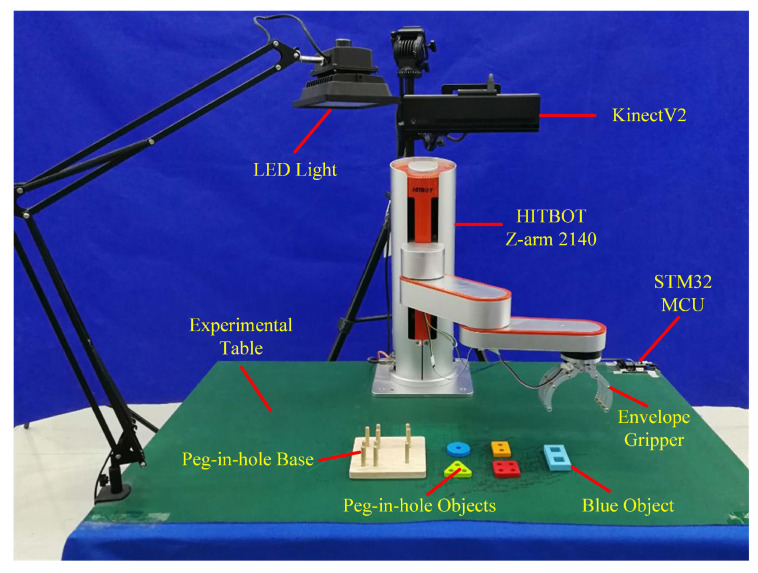
The experimental setup for peg-in-hole assembly tasks.

The 4-DOF SCARA type cooperative industrial manipulator named HITBOT Z-arm 2140 was selected to demonstrate the proposed task-learning strategy with the experimental setup shown in Figure 4. The end-effector was equipped with a 1-DOF gripper, which was driven by a servo motor and controlled by an STM32 microcontroller (MCU) to achieve envelope grasping. Task-relevant objects included a blue object, peg-in-hole objects, and peg-in-hole base. The KinectV2 was mounted on the top with a LED light provided for supplementary illuminations.

### 3.2. Data Acquisition and Learning

**Figure 5:**
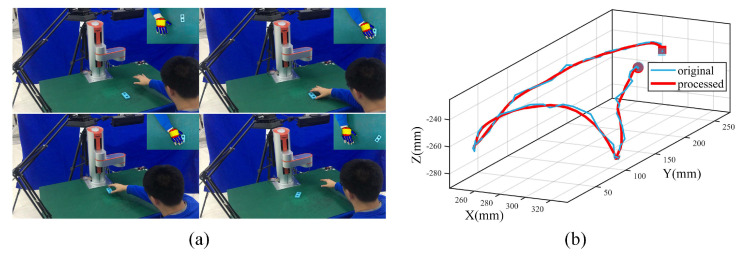
Data acquisition and preprocessing for pick-and-place operations. (**a**) Snapshots of human demonstration with the Kinect FORTH system. (**b**) The original data after calibration with the processed data after Moving Average Filter (MAF) and Cubic Spline Interpolation (CSI).

To learn fundamental trajectory components in peg-in-hole tasks, we demonstrated pick-and-place operations of a blue object 10 times with continuous human hand centroid trajectory coordinates in XYZ positions tracked by the Kinect FORTH system. Original trajectory data were first converted to the robot base frame by hand-eye calibration, then smoothed by MAF with the window width n=4 and finally normalized to 100 timesteps by CSI, as shown in Figure 5. Snapshots in one demonstration are shown in Figure 5a when the human is just about to arrive at the initial point, the pick point, the place point, and the final point defined in Section 2.2. Though there were small deviations between hypothesized 3D hand models and actual human hand observations, they had little effect on tracking human hand centroid trajectories. Original data, after calibration (blue line), and processed data after MAF and CSI (red line) are shown in Figure 5b. The initial point and final point of the complete movements are marked with a dot and square, respectively. Results show that we can track human hand movements accurately and robustly with normalized trajectories of satisfactory smoothness and distortion.

**Figure 6:**
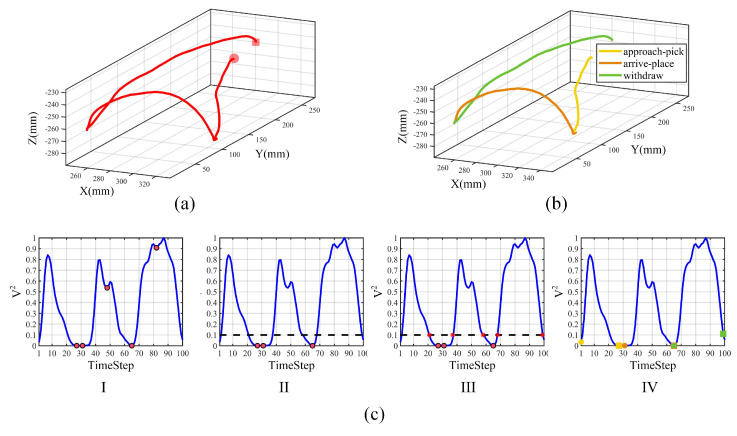
Movement Segmentation results of one demonstration. (**a**) Original pre-processed trajectory. (**b**) Segmented trajectory. (**c**) Movement segmentation process using Algorithm 1. I, II, III, and IV correspond to normalized velocity square sum with local minimum detection, threshold-based elimination, threshold-based intersection, and final segmentation points set, respectively.

Next, we segment each demonstration trajectory into the approach-pick phase, the arrive-place phase and the withdraw phase defined in Section 2.2 using Algorithm 1. Figure 6 shows the movement segmentation results. We first calculated velocities using the Euler approximation at a sample rate of 15 Hz. The calculated velocities were smoothed by MAF twice with the window width n=5 first and n=2 second, then normalized to 100 timesteps by CSI. The square sum of velocities was calculated and normalized within the range $\left[0,1\right]$. We found all the local minimum points and eliminated them using a threshold ϑ=0.1 determined experimentally in 10 demonstrations. Red dots denote local minimum points with black dash line denotes the threshold for elimination. All the intersection points denoted in red asterisks are found between the threshold line and velocity curves. The points were rounded off and updated by determining whether to retain the first intersection point (Algorithm 1, lines 10 to 12). By traversing elements in intersection points set to calculate the distance between intersection points set and local minimum points set using heuristic rules in lines 13 to 24 from Algorithm 1, we can obtain the final segmentation points set. The start points of each phase are denoted in dots with end points marked with squares. The approach-pick phase, the arrive-place phase and the withdraw phase are shown in yellow, orange and green, respectively. Results demonstrate that the proposed heuristic segmentation algorithm can segment continuous human hand centroid trajectories into three phases automatically. The average time of segmentation in 10 demonstrations was 0.26574 s.

**Figure 7:**
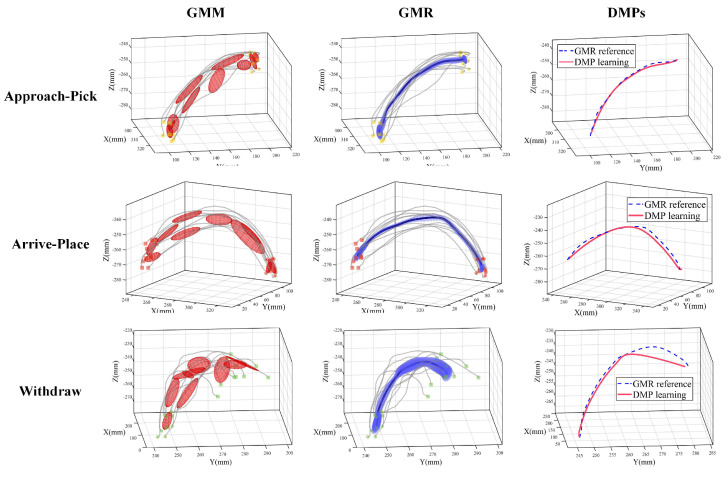
Movement primitives learning results of the approach-pick phase, the arrive-place phase and the withdraw phase in pick-and-place operations. We use Gaussian mixture model (GMM) to encode overall distributions (red ellipsoids) and use Gaussian mixture regression (GMR) to derive the optimal trajectory (blue lines). Dynamical movement primitives (DMPs) are used to learn (red lines) with GMR trajectories as reference (blue dash lines).

After segmentation, we represent three phases in pick-and-place operations with different movement primitives and learn them separately. Figure 7 shows the movement primitives learning results. We adopt the DTW method for time alignment in each phase and select the optimal number *K* of Gaussian components from 1 to 10 based on the BIC score. The overall distributions of trajectories are then denoted by red ellipsoids with GMM. The approach-pick phase, the arrive-place phase and the withdraw phase are represented in yellow, orange and green with start points denoted in dots and end points denoted in squares, respectively. GMR was used to generate the optimal trajectory in 10 demonstrations which encapsulate as many sufficient human features as possible. The generated trajectory of each phase is denoted in blue lines with surrounding shadow areas. In particular, blue lines indicate the expectation of trajectories, and surrounding areas indicate the covariance which reflects restrictions of human hand movements during demonstration, quantitatively. Optimal trajectories with GMR are treated as the reference trajectory for DMPs to learn. We learn weights of 10 basis functions using LWR and demonstrate learning effects by setting start points and end points of DMP the same as GMR, the results are shown in the last column of Figure 7. DMP learning results are shown in red lines with reference GMR trajectories shown in blue dash lines. From Figure 7, we see that the trajectory generated by the DMPs was different from GMR reference in the withdraw-phase. The reason may be that we set the fixed number of Gaussian components with 10 for three phases. The number of Gaussian components was sufficient for the approach-pick phase and the arrive-place phase in that the variations are smaller compared to the withdraw phase. However, for the withdraw phase, the variations are bigger, the number of basis functions was not enough to represent the function *f*, which led to the underfitting problems. Thus, the drawbacks of our method are that DMPs cannot choose appropriate Gaussian components automatically from the data, which needs further improvement. In total, we see that DMPs can retain overall shapes and trends of trajectories though there are small deviations in each phase, making it feasible for robots to achieve trajectory generalization by adapting start points and end points of each phase during task execution.

**Figure 8:**
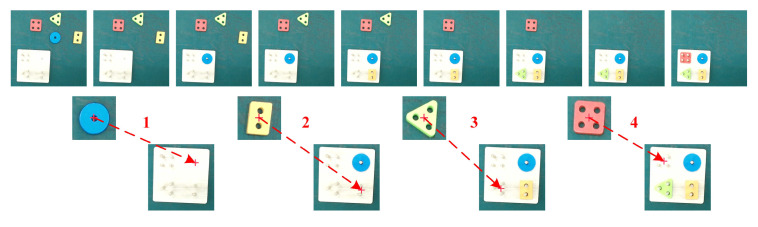
Goal configurations learning results of peg-in-hole tasks from a single demonstration. The first line shows image sequences under the Kinect view via keyframes extraction from the video. The bottom lines are obtained sequences of pre-pick and peg-in-hole observations using the improved VSL algorithm. Red symbols ‘+’ denote the calculated pixels centers with red arrows and numbers represent the task execution order.

Meanwhile, we demonstrate the complete multioperation peg-in-hole tasks only once with the video recorded under the Kinect view for goal configurations learning using the improved VSL algorithm. As shown in Figure 4, task-relevant objects involve four different peg-in-hole objects and one peg-in-hole base. Figure 8 shows the goal configuration-learning results. A sequence of pre-action and post-action images representative for peg-in-hole tasks are obtained from the video by keyframes extraction, as shown in the first line of Figure 8. We utilized image processing methods by first performing image subtraction between adjacent images and converting them to binary images, then conducting morphological erosion and dilation to ensure only one connect domain, finally calculating pixels centers and generating pre-pick observations and peg-in-hole observations defined in Section 2.5. The size of pre-pick observations is 120 × 120 pixels with peg-in-hole observations equal to 300 × 300. Pixels centers are shown in red symbols ’+’ with red arrows and operation numbers represent the task execution order following the temporal relationships of the video. Results show that the improved VSL algorithm can learn goal configurations from only one demonstration with appropriate observations and exact pixel centers obtained to achieve stable and accurate goal configurations generalization under new task situations.

### 3.3. Task Reproduction and Generalization

**Figure 9:**
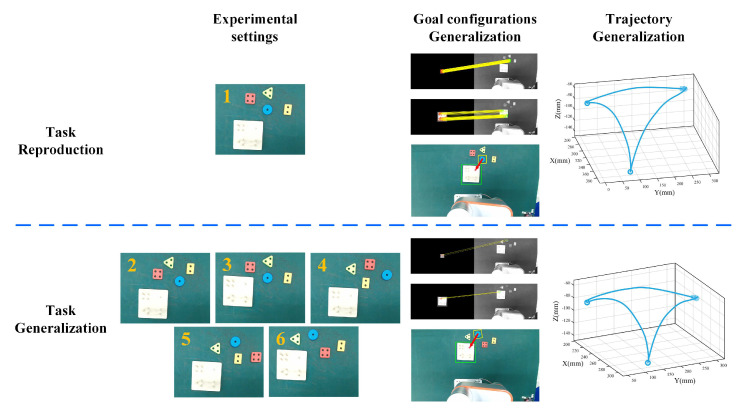
Experimental settings and the generalization process in peg-in-hole reproduction and generalization tasks. Task complexity is gradually increased from Experiment 1 to Experiment 6 (the first column). Taking a blue peg-in-hole object as an example, goal configurations generalization is based on image matching methods with SURF and MSAC algorithm (the second column). The generalized goal configurations are passed to DMPs as query points for trajectory generalization to accomplish peg-in-hole tasks (the last column).

To verify the effectiveness of the proposed task-learning strategy, we conducted six experiments with increased complexity in reproduction and generalization tasks. Figure 9 shows experimental settings and general steps for robots to accomplish peg-in-hole tasks. Experiment 1 was the reproduction task with task-relevant objects placed in the same positions as the goal configurations learning process. Experiments 2–6 were generalization tasks taking account both trajectory and goal configurations generalization. Compared to Experiment 1, Experiments 2 and 3 mainly focus on trajectory generalization with slight position differences of peg-in-hole objects and base respectively. In contrast, Experiments 4 and 5 focus more on goal configurations learning with significant position differences of peg-in-hole objects, and finally Experiment 6 considers the complex cases when goal configurations of peg-in-hole objects and positions of peg-in-hole base change simultaneously.

To illustrate the generalization process, we chose a blue peg-in-hole object in Experiments 1 and 6 as an example. We found all interest matched feature points using the SURF algorithm [36], then rejected unreliable matching pairs and calculated the homography matrix with the MSAC algorithm [37]. The calculated homography matrix was used to transform pixels center of learned goal configurations to the image plane under new task situations, as shown in the second column of Figure 9. Red symbols ‘+’ denote the calculated pixel centers of the image plane with the task execution order shown in red arrows. The observations of objects (120×120 pixels) and base (300×300 pixels) are shown in yellow and green, respectively. We see that there are obviously more feature points matched in the reproduction task compared to the generalization task, the reason may be that images exhibit more similarity in the reproduction task. However, we can still obtain exact pixel centers using only a small amount of matched feature points based on the reliable estimation of MSAC algorithm. Generalized goal configurations under the image plane are then transformed to 3D actual pick-and-place points under the robot base frame by hand-eye calibration with pre-pick offsets and peg-in-hole offsets determined, further passed to DMPs as query points for trajectory generalization to accomplish peg-in-hole tasks, as shown in the last column of Figure 9. The initial and end points (denoted in blue dots and squares) were set the same, and the pick-and-place points (denoted in hollow blue dots) were updated according to the generalized goal configurations.

**Figure 10:**
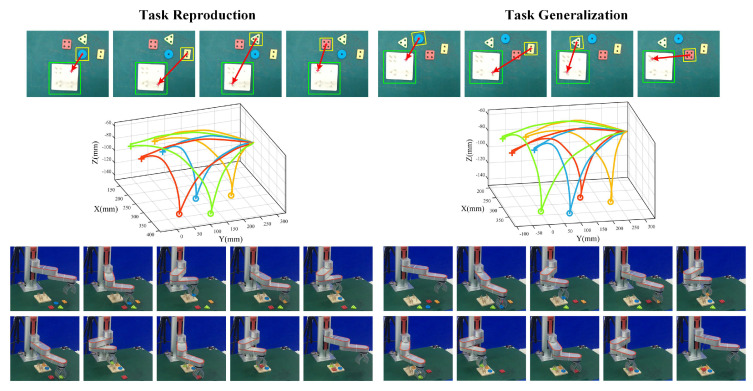
Complete execution process of peg-in-hole tasks in the reproduction task (Experiment 1) and the generalization task (Experiment 6). The first line denotes goal configurations generalization results, and the second line represents trajectory generalization results. Symbols ‘o’ and ‘+’ denote the actual pick-and-place points with different colors distinguished. The bottom line shows snapshots of the robot execution process.

Figure 10 shows the complete execution process of peg-in-hole tasks. Due to the lack of space, we just provided results in Experiments 1 and 6 representative of the reproduction task and generalization task. Goal configurations generalization results of four peg-in-hole objects and one base following the task execution order are shown in the first line. We see that the improved VSL algorithm was able to obtain the exact pick-and-place points under the image plane. After hand-eye calibration with pre-pick offsets and peg-in-hole offsets added, we acquire actual 3D pick-and-place points under the robot base frame and pass to learned DMPs for trajectory generalization with results shown in the second line. Symbols ‘o’ and ‘+’ denote the actual pick-and-place points, and the whole trajectories are distinguished by different colors of peg-in-hole objects (blue, orange, green, red, respectively). Results demonstrate that the generated smooth trajectories in Experiments 1 and 6 have a high similarity, and snapshots of the robot execution process are shown in the bottom line including fixed initial points and final points as well as different pick-and-place points to accomplish peg-in-hole tasks.

## 4. Result and Discussion

In this section, in order to illustrate the benefit and performance of the proposed task-learning trajectory, we analyze the experimental results from four aspects: movement segmentation, trajectory generalization, goal configurations generalization, robustness of the algorithm, and summarize the advantages and limitations of the proposed strategy in the end.

### 4.1. Movement Segmentation Performance

**Figure 11:**
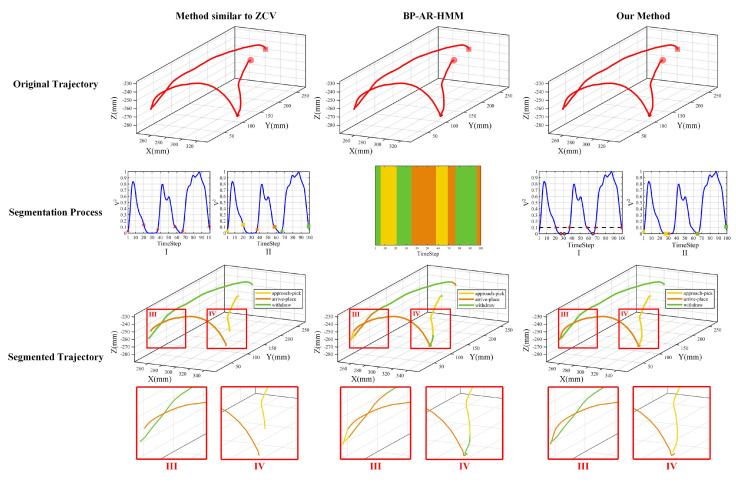
Comparison results of the movement segmentation performance in one demonstration. The first row: the input original trajectory passed to the method similar to zero-crossing velocity (ZCV) [12] (the first column), Beta Process Autoregressive Hidden Markov Model (BP-AR-HMM) [26] (the second column), and our method (the last column). The second row: the segmentation process of three methods. The last row: the segmentated trajectories of different phases in peg-in-hole tasks.

Specific to the performance of the movement segmentation algorithm, we compared our method with typical heuristic methods similar to ZCV in [12] and typical unsupervised learning methods like BP-AR-HMM in [26]. Following ZCV methods in [12], we selected the threshold ϑ=0.1 and assigned υ=0 if the sum of velocities was below the value ϑ, and υ=1 in the opposite case. If $\upsilon \left(i\right)=0$ and $\upsilon \left(i+1\right)=1$, the timestep *i* is treated as a rising edge, and if $\upsilon \left(i\right)=1$ and $\upsilon \left(i+1\right)=0$, the timestep *i* is treated as a falling edge. Then timesteps between rising edges and falling edges were used to construct segmentation points for different phases in peg-in-hole tasks. Subsequently, according to BP-AR-HMM methods in [26], we first preprocessed multiple demonstrations so that the variance in the first differences of each dimension is 1, and the average value is 0. We set the same BP-AR-HMM parameters in [26] and ran 10 Metropolis-Hastings and Gibbs sampler with 1000 iterations each, producing 10 segmentations. Then, segmentation results with the highest log likelihood are obtained during the iteration process. The comparison results of three methods are shown in Figure 11, and we calculate the total segmentation time, the average segmentation time, and the standard deviation of three methods in 10 demonstrations, the results can be seen from Table 1.

In Figure 11, the first row shows the input original trajectory passed to the method similar to ZCV [12] (the first column), BP-AR-HMM [26] (the second column), and our method (the last column), respectively. The initial points and final points are marked with dot and square. The second row shows the segmentation process of three methods. In the method similar to ZCV, rising edges (asterisk) and falling edges (triangle) are shown in the velocity curve I with II the obtained segmentation points. The start points (dot) and end points (square) are shown in yellow (the approach-pick phase), orange (the arrive-place phase) and green (the withdraw phase), respectively. In BP-AR-HMM, the demonstration is marked with different color bars. In our method, the velocity curve I denotes the heuristic segmentation process based on intersections (asterisk) and local minimums (dot), and II corresponds to the obtained segmentation points. Finally, the last row shows the segmented trajectory with segmentation points indexed to acquire trajectories of different phases. In order to illustrate the movement segmentation results better, local enlarged images are added. Specifically, III and IV denote the areas around the connections of the arrive-place phase and the withdraw phase, the approach-pick phase and the arrive-place phase, respectively.

From Figure 11 and Table 1, we see that the method similar to ZCV can achieve fast segmentation, however, the segmentation results are sub-optimal and lose essential features of human demonstrations. It may lead to an insufficient movement primitives learning process that encapsulates fewer features of human demonstrations. The BP-AR-HMM method needs a large amount of training time. In addition, though BP-AR-HMM can achieve automatic segmentation with minimum prior knowledge, trajectories of different phases are mixed in this case, the reason may be that the noise interference of the vision capture system and similar motion characteristics exhibited in different phases of pick-and-place operations which have little distinctions between each other. In contrast, our method takes a longer time compared to the method similar to ZCV, but we can obtain better segmentation points encapsulating as sufficient features as possible to facilitate movement primitives learning. However, our method only aims at the identical task type, when facing different types of tasks, the performance of our method will not match the BP-AR-HMM method.

### 4.2. Trajectory Generalization Performance

In this paper, there are six peg-in-hole experiments (see Figure 9), and peg-in-hole tasks in one experiment include four operations of blue, orange, green, red objects in turn. Based on that, we considered four peg-in-hole operations as four subtasks named subtask 1, subtask 2, subtask 3, subtask 4, respectively. To quantitatively evaluate the trajectory generalization performance, we considered the trajectory similarity measure and trajectory smoothness measure. Specifically, the concatenated human demonstration trajectory in different phases generated by GMR (see Figure 7) is treated as the reference trajectory, and we calculate the trajectory similarity and trajectory smoothness for each subtask in each experiment. According to [38], we adopt the cosine similarity measure by computing the cosine similarity between each segment of trajectories and normalize the results with the range $\left[0,1\right]$ as follows:

(13) $$M\_similarity=\frac{1}{T-1}\sum_{t=1}^{T-1}\frac{\left(x_{t+1}-x_{t}\right).\left(x_{t+1}^{*}-x_{t}^{*}\right)}{∥x_{t+1}-x_{t}∥∥x_{t+1}^{*}-x_{t}^{*}{∥}^{’}}$$

where M_similarity denotes the calculated trajectory similarity, $x_{t}$ is the generalization trajectory vector with $x_{t}^{*}$ the demonstration trajectory vector generated with GMR, *T* is the total timesteps of the generalized trajectory. Generally, calculated trajectory similarity close to 1 indicates that the generalized trajectory has a high similarity relative to the human demonstration trajectory. Table 2 shows the trajectory similarity measure results, we can see that the reproduction task (Experiment 1) and generalization tasks (Experiments 2 to 6) exhibit high similarity compared to the human demonstration trajectory.

Meanwhile, we calculate the derivation of the acceleration extracted from the motion according to [39] as follows:

(14) $$M\_smoothness=\frac{1}{T-3}\sum_{t=1}^{T-3}∥{\dddot{x}}_{t}∥,$$

where M_smoothness denotes the calculated trajectory smoothness, ${\dddot{x}}_{t}$ is the derivation of the acceleration based on the differential process, *T* is the total timesteps of the generalized trajectories. In total, calculated trajectory smoothness close to 0 indicates that the generalized trajectory has a high smoothness. Table 3 shows the trajectory smoothness measure results, it can be seen that compared to the demonstration trajectory, the trajectory smoothness is reduced in the reproduction task (Experiment 1) and the generalization tasks (Experiments 2 to 6). However, it exhibits satisfactory smoothness in an acceptable range to accomplish peg-in-hole tasks.

Subsequently, in order to further demonstrate the trajectory performance of the proposed strategy, we compare our method using GMM-GMR and DMP to other trajectory planning methods using quintic polynomial splines [40]. Trajectory planning methods based on quintic polynomial splines are methods suitable for solving the velocity jerky and acceleration jumping problems in robotics. To generate trajectories for comparison, the generalized points of subtask 1 to 4 in six experiments were treated as the boundary position conditions with velocities and accelerations constrained to zero. We calculated the trajectory similarity using Equation (13) and the trajectory smoothness using Equation (14). The results can be seen from Table 4 and Table 5, with the average and standard deviation of trajectory similarity and smoothness in two methods shown in Table 6. From Table 4, Table 5 and Table 6, we see that our method using GMM-GMR and DMP exhibits higher similarity but lower smoothness compared to trajectory planning methods using quintic polynomial splines. Thus, our method incorporates sufficient human features into the generalized trajectories but loses partial smoothness. Note that in this paper, the trajectory smoothness is in an acceptable range for the robot to accomplish peg-in-hole tasks.

### 4.3. Goal Configurations Generalization Performance

In this paper, goal configurations refer to the actual pick-and-place points in peg-in-hole tasks generalized from the improved VSL algorithm, as a consequence, the success rate of peg-in-hole tasks indirectly reflects the goal configurations generalization performance. In order to evaluate the task success rate, we consider each experiment (see Figure 9) consists of four sub-tasks (blue, orange, green, and red objects, respectively), and the sub-task is considered correct if the robot executes peg-in-hole tasks successfully and failed if the robot does not grasp or release objects correctly during task execution. The success rate of peg-in-hole tasks can be seen in Table 7. Totally, there are 4 filed tasks in 24 subtasks of 6 experiments with a success rate of 83.3%. Note that although we can obtain exact pick-and-place points using the improved VSL algorithm, failures sometimes occur owing to uncertainties of calibration errors or gripping errors. If these errors are out of the range allowed by clearances on the holes, the robot can not execute tasks successfully. To date, the proposed task-learning strategy does not have the ability of automatic error recovery [41] and the ability of real-time obstacle avoidance [42], which needs further improvement in future work.

To further demonstrate the goal configurations generalization performance, we compared our method to other related works in [12] which proposes a learning strategy for car assembly operations, and the comparison results can be seen from Table 8. Trajectory learning and generalization are achieved by TP-GMMs in [12], and goal configurations generalization under new task situations is performed by the image identification method based on colors. Two types of experiments are conducted to validate the goal configurations generalization performance. In the first experiment, the positions of task-relevant objects are at restricted areas with a total success rate of 87.7%, and in the second experiment, the restriction is not fulfilled with a total success rate of 63.7%. In contrast, the positions of task-relevant objects in this paper are not restricted strictly (see Figure 9) to verify the goal configurations performance, and the total success rate is 83.3%. From Table 8, we see that our method has a higher success rate compared to the unrestricted case and a lower success rate compared to the restricted case, which to some extent leads to the generality for different task situations. However, we only focus on the fundamental peg-in-hole tasks in this paper with lower task complexity, and the goal configuration generalization performance needs to be verified in more complex tasks in future work.

### 4.4. Robustness of the Algorithm

In this paper, failures in peg-in-hole tasks owing to the uncertainties of calibration errors or grasping errors. Grasping errors mean that after the robot grasps the object, the positions of objects will not be the same all the time, and calibration errors mean that the actual pick-and-place points under the Kinect frame will not transform to the actual 3D pick-and-place points under the robot base frame accurately. If these errors are out of the range allowed by clearances on the holes, the robot cannot execute peg-in-hole tasks successfully. With regard to the robustness of the algorithm, in this paper, the clearances of holes allow the peg-in-hole operations do not require an exact estimation, as a result, the robustness of the algorithm is ensured by appropriate clearances on the holes. However, for peg-in-hole tasks with higher precision, the clearances will not allow the uncertainties of calibration errors or grasping errors, leading to poor robustness of the algorithm. Compared to other learning strategies like [43] which combine force/moment information to determine the contact states of peg-in-hole tasks with reinforcement learning, our algorithm does not have the robustness upon high precision peg-in-hole tasks, which needs further improvement in future work.

### 4.5. Advantages and Limitations of the Proposed Strategy

To summarize, this paper proposes a novel task-learning strategy aiming at fundamental peg-in-hole tasks in robotic assembly. The major advantages of the strategy include: (1) we can acquire continuous human hand movements flexibly during demonstration based on the markerless vision capture system, which allows extensions to different robotic manipulators; (2) we can automatically segment the complete human movements into different phases represented by different movement primitives, the optimal trajectory encapsulating sufficient human features is generated with GMM-GMR, and learned with DMPs to endow the robot the ability of trajectory generalization under new task situations; (3) we can learn goal configurations directly from the video of the human multioperation demonstration and generalize goal configurations as exact actual pick-and-place points under new task situations based on the improved VSL algorithm.

However, there are also limitations for the proposed task-learning strategy: (1) the experiment setup in this paper only considers the controlled lab conditions, far from the real-world applications. For real world scenarios with bad illumination conditions and unstructured dynamic enviroments, the task-learning strategy may exhibit a poor performance in that the quality of images captured with the vision capture system are not as good as the lab, leading to inaccurate trajectory data and inexact pick-and-place points generalization. (2) The peg-in-hole tasks investigated in this paper are simple tasks, for relatively complex set of tasks like parts assembly, the task-learning strategy may exhibit poor performance, the reason may be that the robot needs to determine the actual task-relevant goals in real-time during task execution, if occlusions occurred by the human body or the robot body, the robot will not find the correct task-relevant goals based on the visual learning method. (3) In this paper, the success rate of peg-in-hole tasks is ensured by appropriate clearances on the holes, however, when implementing the strategy in the area of industrial HRC, where the precision of peg-in-hole tasks is usually high, merely relying on vision positioning is not enough to accomplish the tasks, the success rate may have a poor performance hence. (4) Given that in a real-world industry, there are different semi-skilled workers for their day-to-day tasks, the workers may not be able to demonstrate the robot as perfectly with their hand gestures as is done by a well-trained sole experiment subject in the lab, as a result, the proposed strategy needs to be verified with 15 to 20 human subjects to carry out the full-scale subjective evaluation of the LfD system when implementing it in the HRC area.

## 5. Conclusions

This paper presents a novel task-learning strategy that integrates goal configurations learning and trajectory learning. For trajectory learning, we track continuous human hand centroid movements using the Kinect FORTH system with a threshold-based heuristic movement segmentation algorithm proposed to segment trajectories into the approach-pick phase, the arrive-place phase and the withdraw phase. We represent three phases with different movement primitives and learn them separately. GMM-GMR is used to extract the optimal demonstration trajectory with sufficient human features and DMPs are utilized to learn. Trajectory generalization is achieved by adapting start points and end points of each phase with DMPs. Meanwhile, we develop an improved VSL algorithm to learn goal configurations visually from only one human demonstration of multioperation peg-in-hole tasks. We define peg-in-hole observations and offsets for adaption to peg-in-hole tasks. Goal configurations generalization is achieved by SURF and MSAC algorithm with coordinate transformation performed to obtain actual 3D pick-and-place points under the robot base frame. After learning, robots can generalize the actual pick-and-place points under new task situations and generate complete trajectories for task execution. The performance of the task-learning strategy is validated on a 4-DOF SACRA type HITBOT manipulator in six peg-in-hole experiments with gradually increased task complexity, and the total success rate is 83.3%. Experimental results show that robots can obtain the exact pick-and-place points and generate trajectories with satisfactory similarity and smoothness. Moreover, the proposed strategy allows extensions to different robotic manipulators thanks to the visual learning methods.

However, in this paper, the robot is not equipped with the ability for real-time obstacle avoidance during task execution, nor the ability to increase the task success rate by self-learning. In addition, the experimental setup only considers the controlled lab conditions, far from real-world applications. As a result, future work will focus on the following directions: (1) a modified trajectory learning method based on ProMPs to achieve via-points generalization for real-time obstacle avoidance; (2) integrate some reinforcement learning methods into the strategy to increase the task success rate; and (3) validate and improve the task-learning strategy in real-world applications.

## Figures and Tables

**Table 1:** Segmentation time results of three methods in 10 demonstrations.

	Method Similar to ZCV	BP-AR-HMM	Our Method
**Total time (s)**	0.0127	3551.0065	2.6574
**Average time (s)**	0.00127	355.10065	0.26574
**Standard Deviation (s)**	0.0015	3.5492	0.0350

**Table 2:** Trajectory similarity measure in robotic peg-in-hole tasks.

	Experiment 1	Experiment 2	Experiment 3	Experiment 4	Experiment 5	Experiment 6
Subtask 1	0.9147	0.9181	0.9054	0.9095	0.9166	0.9115
Subtask 2	0.9066	0.9008	0.9081	0.9025	0.9067	0.9054
Subtask 3	0.9007	0.9004	0.9088	0.8992	0.8997	0.9007
Subtask 4	0.9042	0.9111	0.9119	0.9112	0.8853	0.8928

**Table 3:** Trajectory smoothness measure in robotic peg-in-hole tasks. (The calcualted smooth measure of human demonstration is 0.0648).

	Experiment 1	Experiment 2	Experiment 3	Experiment 4	Experiment 5	Experiment 6
Subtask 1	0.1003	0.1027	0.1084	0.1219	0.1117	0.1117
Subtask 2	0.1246	0.1300	0.1348	0.1099	0.1140	0.1371
Subtask 3	0.1422	0.1395	0.1478	0.1341	0.1317	0.1285
Subtask 4	0.1182	0.1119	0.1202	0.1334	0.1502	0.1342

**Table 4:** Trajectory similarity measure for quintic polynomial splines method [40].

	Experiment 1	Experiment 2	Experiment 3	Experiment 4	Experiment 5	Experiment 6
Subtask 1	0.8620	0.8693	0.8553	0.8697	0.8731	0.8639
Subtask 2	0.8687	0.8637	0.8683	0.8561	0.8662	0.8690
Subtask 3	0.8714	0.8681	0.8780	0.8618	0.8653	0.8625
Subtask 4	0.8600	0.8654	0.8698	0.8768	0.8424	0.8499

**Table 5:** Trajectory smoothness measure for quintic polynomial splines method [40].

	Experiment 1	Experiment 2	Experiment 3	Experiment 4	Experiment 5	Experiment 6
Subtask 1	0.0044	0.0043	0.0048	0.0044	0.0046	0.0053
Subtask 2	0.0048	0.0052	0.0117	0.0048	0.0046	0.0052
Subtask 3	0.0064	0.0064	0.0132	0.0129	0.0063	0.0067
Subtask 4	0.0059	0.0056	0.0062	0.0057	0.0054	0.0058

**Table 6:** Comparison results of trajectory generalization between quintic ploynomial splines method [40] and our method.

	Quintic Method	Our Method
**Average Similarity**	0.8648	0.9055
**Standard Deviation**	0.0026	0.0026
**Average Smoothness**	0.0161	0.1250
**Standard Deviation**	0.0123	0.0035

**Table 7:** The success rate in robotic peg-in-hole tasks.

Task	Correct	Failed	Total	Success Rate
Subtask 1 (blue)	5	1 (experiment 5)	6	83.3%
Subtask 2 (orange)	5	1 (experiment 3)	6	83.3%
Subtask 3 (green)	5	1 (experiment 2)	6	83.3%
Subtask 4 (red)	5	1 (experiment 6)	6	83.3%
**Total**	**20**	**4**	**24**	**83.3%**

**Table 8:** Comparison results of goal configurations generalization between image identification method [12] and our method.

	Image Identification Method [12]	Our Method
**Success Rate**	Experiment 1: **87.7%** and Experiment 2: **63.4%**	**83.3%**
